# Long COVID in healthcare workers: longitudinal mixed-methods study

**DOI:** 10.1093/occmed/kqae113

**Published:** 2024-11-29

**Authors:** A Grant, N N Adams, E MacIver, D Skåtun, N Scott, C Kennedy, F Douglas, V Hernandez-Santiago, N Torrance

**Affiliations:** School of Health, Robert Gordon University, Aberdeen AB10 7QB, UK; School of Health, Robert Gordon University, Aberdeen AB10 7QB, UK; School of Health, Robert Gordon University, Aberdeen AB10 7QB, UK; Health Economics Research Unit, University of Aberdeen, Aberdeen AB25 2ZD, UK; Division of Applied Health Sciences, University of Aberdeen, Aberdeen AB25 2ZD, UK; School of Pharmacy, Applied Sciences and Public Health, Robert Gordon University, Aberdeen AB10 7QB, UK; School of Health, Robert Gordon University, Aberdeen AB10 7QB, UK; School of Medicine, University of St Andrews, St Andrews KY16 9TF, UK; School of Health, Robert Gordon University, Aberdeen AB10 7QB, UK

## Abstract

**Background:**

Healthcare workers (HCWs) report higher rates of long coronavirus disease (COVID) (LC) than other occupational groups. It is still unclear whether LC is a lifelong condition. Workforce shortfalls are apparent due to sick leave, reduced hours and lower productivity.

**Aims:**

To investigate the lived experience of LC on a range of HCWs, including impact on health-related quality-of-life (HRQL), use of health services, working and personal lives and household finances.

**Methods:**

Longitudinal mixed methods with online surveys and qualitative interviews 6-months apart. HCWs including healthcare professionals, ancillary and administration staff who self-report LC were recruited through social media and National Health Service channels. Interviewees were purposively sampled from survey responses.

**Results:**

The first survey was completed by 471 HCWs (S1) and 302 (64%) the follow-up (S2). A total of 50 HCWs were interviewed initially and 44 at second interview. All participants experienced various relapsing, remitting, changing and prolonged LC symptoms (mean 7.1 [SD 4.8] at S2) and a third reported day-to-day activities ‘limited a lot’. Most participants were working in a reduced capacity: reduced hours, different role or location. Healthcare was limited, and often unsatisfactory. Participants feared reinfection, their future, ability to work and financial security (59% (*n* = 174) at S2). They experienced stigma, distress, grief for their former self and some felt unsupported, however, as awareness of LC grew some experienced improved understanding and support.

**Conclusions:**

Most participants continued working, managing complex and dynamic symptoms effecting their everyday life and ability to work. Most did not report significant improvements over time and feared for their future and financial security.

## Teaser

Healthcare workers report higher rates of long coronavirus disease (COVID) (LC) than other occupational groups. It is still unclear whether LC is a lifelong condition. We investigated the lived experience of healthcare professionals and ancillary staff with self-reported LC over time. All participants experienced a range of relapsing-remitting and changing symptoms which affected their everyday life and ability to work. Exacerbations were associated with reinfection, vaccination and unsuccessful returns to work. All were concerned about their finances and future.

## Introduction

Long coronavirus disease (COVID) (LC, or post-acute sequelae of COVID-19) is a condition associated with multifaceted and fluctuating symptoms for 12 weeks or more following COVID-19 infection that cannot be explained by another diagnosis. Over 200 symptoms are associated with LC, the most common are fatigue, headache and brain fog [1]. It is estimated 65 million people worldwide have LC based on observational data [2]. However, this is likely to be higher due to issues with diagnosis and heterogeneity in prevalence estimates [3]. The LC population is dynamic, with reinfection of COVID-19 increasing the risk of developing LC [4]. The European Commission estimate that LC caused a workforce shortfall of 0.2–0.3% in 2021–22 through sick leave, reduced hours, lower productivity or inactivity [5].

Healthcare workers had higher occupational risk of exposure to COVID-19 than the general population [6–8] and report higher incidence of LC [9]. The working-age group has the highest risk of developing LC [10] and the debiliating effects impact ability to work, it is estimated 10 000 healthcare staff in the UK are off work with LC [11]. A British Medical Association (BMA) survey of doctors with LC found 18% were unable to work, 31% were in working part-time (compared with 57% pre-pandemic) and 48% experienced loss of earnings [12].

The Nursing and Midwifery Council and the BMA have written to the UK government calling for LC to be recognized as an occupational illness, which would mean those who have worked in healthcare and suffering long-term would be eligible for Industrial Injuries Disability Benefit [13]. A group of doctors have launched a legal case against the National Health Service (NHS) for compensation after contracting COVID-19 at work and developing LC, which has devastated their lives and affected their ability to work, with some forced to sell their homes and use foodbanks [14].

The NHS is experiencing a workforce crisis, with unprecedented demand for services, high levels of vacancies and burnout [15,16]. There is a substantial rise in the number of people of working age unable to work or limited in what work they can do due to long-term illness [17].

This longitudinal study aimed to investigate the lived experience of the longer-term effects of COVID-19 on HCWs. Data were collected by online survey and in-depth qualitative interview, with follow-up after 6 months. We asked about symptoms, health-related quality-of-life (HRQL), access and use of health services, impact on working lives and finances [18].

## Methods

A longitudinal mixed-methods study comprising an online questionnaire survey of NHS workers with self-reported symptoms of LC. Information about the study was disseminated via online support groups, social media and NHS boards. In-depth qualitative interviews were then conducted with a sample of 50 of these NHS workers. Six months later, participants were invited to complete a follow-up survey and (if applicable) interview. All data were collected between June 2021 and August 2022.

The online questionnaire asked about symptoms and used validated health and wellbeing scales [18]. HRQL was assessed using the Medical Outcomes Short Form-12 (SF-12) [19], and EuroQol five-dimensional five-level questionnaire (EQ-5D-5L) [20]. Information on mental wellbeing, depression and anxiety symptoms was collected using the Warwick-Edinburgh Mental Wellbeing Scale (WEMWBS) [21] and the Patient Health Questionnaire (PHQ-4) [22]. Fatigue was assessed using the Promis SF-V1-4A Measure of Fatigue [23]. Descriptive analyses were conducted to examine any changes over the 6-month period, including paired *t*-tests for continuous data and McNemar tests for binary categorical data. *P* < 0.05 was considered statistically significant. All questionnaire data were analysed using Stata v17.

Interviewees were purposively sampled for maximum variation in occupations, sociodemographic characteristics (as Table 1) and severity of LC based on survey responses. Interviews were conducted primarily via Microsoft Teams, with a small number via telephone, by A.G., E.M. and N.A., and lasted between 30 and 120 minutes (I1: Mean: 50 minutes; Median: 44 minutes. I2: Mean: 42 minutes; Median: 40 minutes). Qualitative data analysis utilized Braun and Clark [24]. More detail on the methods can be found in Torrance *et al.* [18].

**Table 1.** Interviewee demographic information

	Ancillary admin/other	Allied health professional	Medical doctor	Nurse	Total I’view 1	Total I’view 2
BAME^b^	1	2	2	1	6	6
Age ≤ 25	0	0	0	2	2	2
26–35	1	2	1	3	7	7
36–45	4 (3)^a^	3	5	1	13	12
46–55	2	3	3 (1)	11 (10)	19	16
56–65	3 (1)	2	1	2	8	6
66+	0	0	0	1	1	1
Male	2	1	3	2	8	8
Female	8 (5)	9	7 (5)	18 (17)	42	36
Primary care	1	1	4	6	12	12
Secondary care	9 (6)	9	6 (4)	14 (13)	38	32
I’view 1 Total	10	10	10	20	50	–
I’view 2 Total	7	10	8	19	–	44

^a^Bracketed numbers refer to interview two.

^b^BAME refers to Black, Asian and Minority Ethnic groups.

Ethical approval was from Robert Gordon University (Ref 21-04) and NHS Research and Development management approvals were obtained from all NHS Scotland Health Boards (IRAS: 298496).

## Results

A total of 471 NHS workers completed the first survey (S1) and 302 (64%) the follow-up (S2). Fifty participants were interviewed following S1, and 44 (88%) participants took part in the follow-up interviews. One participant is known to have died from the effects of LC.

At S1 and S2, participants reported similar sociodemographic and professional characteristics; 98% reported their ethnicity as White; 60% worked in a hospital (Table 2). Sixty-four per cent had a COVID-19 test (*n* = 298) when they first had any symptoms of acute infection.

**Table 2.** Sociodemographic characteristics of survey respondents at both time points

	Survey 1 (*n*(%) = 471)	Follow-up (S2) (*n*(%) = 302)
Age		
Under 25 years	19 (4)	9 (3)
26–35 years	80 (17)	41 (14)
36-45 years	124 (26)	71 (24)
46–55 years	153 (33)	112 (37)
>56 years	93 (20)	64 (21)
Gender		
Female	431 (92)	278 (92)
Male	35 (7)	21 (7)
Prefer not to say	5 (1)	3 (1)
Ethnicity		
White	456 (98)	296 (98)
Non-white	9 (2)	6 (2)
Occupational group		
Nurse & midwives	226 (48)	151 (50)
Doctor	37 (9)	24 (8)
Allied health professional	52 (11)	28 (9)
Ancillary	51 (11)	37 (12)
Administrative	79 (17)	45 (15)
Other	26 (6)	17 (6)
Work setting		
Hospital	282 (60)	174 (58)
Community	129 (27)	84 (28)
Other location	59 (13)	43 (14)
Hours worked		
Full-time (37.5 hours/week or more)	244 (52)	160 (53)
Part-time (21–37.5 hours/week)	176 (37)	112 (37)
Part-time (<21 hours/week)	33 (7)	19 (6)
No longer working (unable, retired/N/K)	18 (4)	11 (4)
Smoking status		
Non smoker	341 (74)	213 (72)
Current smoker	21 (5)	13 (4)
Previous smoker	102 (22)	70 (24)
Frequency of LC symptoms		
All of the time	148 (32)	67 (23)
Most of the time	205 (44)	106 (36)
Some of the time	93 (20)	90 (30)
Occasionally	23 (5)	26 (9)
None of the time	N/A	7 (2)

Demographic information for the participants in the qualitative interviews is shown in Table 1.

All participants experienced a range of relapsing-remitting and changing symptoms that affected their everyday life. The mean number of LC symptoms reduced from 9.5 [SD 5.2] at S1 to 7.1 [SD 4.8] at S2. The most common and debilitating symptoms were fatigue (73%), ‘brain fog’ (70%) and breathlessness (54%) at S2, with a slight decrease in the proportion reporting these between surveys (full list in Table 1, available as Supplementary data at *Occupational Medicine* online). 58% (*n* = 173) reported symptoms ‘all/most of the time’ at S2, compared to 75% at S1 (*n* = 353). However, only seven (2.4%) participants reported no LC symptoms at S2.

Overall, there was a reduction in those whose day-to-day activities were ‘limited a lot’ (51% (*n* = 241) at S1 to 32% (*n* = 89) at S2 (Figure 1, available as Supplementary data at *Occupational Medicine* online), although 84% (*n* = 236) still had some activity limitations at S2 (i.e. only 16% reported NO limitations after 6 months, *n* = 44).

Participants were highly motivated to recover and engaged in many diverse activities to attempt recovery (medications, supplements, exercise, healthcare seeking, seeking alternative and private healthcare). At interview one (i1), they were experiencing feelings of abandonment, stigma, isolation, depression, anxiety, grief and loss for their previous life and former self. However, by interview two (i2), some participants were more accepting of their current health status and forgoing their previous life (usually social activities). This was key to coping and, in some cases, improvement along with workplace adjustments and being believed by general practitioner (GP) and/or at work and home. However, they still reported grief for their former self and fear of what the future might hold, often asking, ‘will I ever get better?’ Interviewees reported that rest did not improve symptoms but provided temporary relief and time to plan and strategize activities. Fear of reinfection was prevalent across all participants, with exacerbations often associated with reinfection, vaccination and unsuccessful returns to work. Table 4 presents supporting qualiative quotes.

**Table 4.** Qualitative illustrative quotes

Theme description	Illustrative quote
Relapsing and remitting symptoms	‘…I’ve got more symptoms, and they seem to last longer, as well…And also, the memory and concentration’s not great…’ Participant 2, female, 56–65 years old, nurse, Interview 2
Self-management strategies	‘It was just trying to get my head round what I was able to do…I had to be a bit more strategical with my energy levels…’ Participant 1, female, 26–35 years old, AHP, Interview 1
Fear of ability to work, future and financial security	‘…I worry about the future; I think how long can I keep working at a reduced level … I worry that what if the cognitive issues become worse? … what if I can’t work, I don’t know anything else … I worry that if I don’t do that, what else can I do? … if I don’t work as a GP, like I’ve always done, then would we be able to afford the house, would we be able to afford the children’s schools..’. Participant 11, female, 36–45, medic, Interview 1
Acceptance of their current health status and forgoing previous life	‘… that’s been the biggest thing, actually. It’s helped everything else move to a place that I can now see, and just accepting that on a day-to-day basis, it will be different. And some days, I will have a good day and some days I won’t..’. Participant 13, female, 26–35 years old, nurse, Interview 2
Rest not improving symptoms	‘… it’s frustrating, (be)cause they tell you to rest, but I’ve just been resting and I’m not getting any better’. Participant 3, female, 36–45 years old, medic, Interview 2
Invisible symptoms and stigma	‘…they are all invisible symptoms … that’s what’s wrong … (they) think oh come on, you know, you’re not really ill, you could go out and get a job, you’re no really stressed … they’re just wanting some time off their work’. Participant 28, female, 46–55 years old, admin, Interview 2
Health care access	‘… my GP’s very nice, but doesn’t really have much to offer and always ask me if I’ve got any ideas of anything’. Participant 3, Medic, Female, aged 36-45, Interview 1.
Access to occupational health	‘…they called us heroes … when things were bad, but now there’s, there’s nothing and even the occupational, access to occupational health, has been really hard’. Participant 27, female, 36–45 years, AHP, Interview 1
Being believed	‘… It went from not being really believed or heard, or treated to, oh my God, you know, I think the shock on her face when I came in, because I was just so ill, you know, I could hardly walk’. Participant 7, female, 46–55 years old, nurse, Interview 2
Workplace support	‘…I tried to keep going [at work] for over a year … on several occasions, I broke down in tears in meetings with them … citing how tired I was and how much I was struggling, and nobody ever came to me and said, are you sure you should be here? … since I’ve been off their, their communication with me has been non-existent’. Participant 49, male, 36–45 years old, GP, Interview 2
Changing roles/responsibilities	‘…there was a job that came up in [a different specialism] … the pace of things is slower … I reduced my hours as well … which is working really well just to recover’. Participant 27, female, 36–45 years old, AHP, Interview 2

HRQL, mental wellbeing and fatigue scores are shown in Table 3. Between S1 and S2, there were small increases/improvements in mean SF-12 Physical and Mental Component Scores and in EQ-5D-5L Visual Analogue Scale (VAS) scores (from mean 61.5 (SD 19.5) to 63.8 (SD 20.0), *P* = 0.03). There was a small non-significant increase in mean mental wellbeing with WEMWBS scores, from 42.4 (SD 10.2) to 43.1 (SD 10.5). Almost a third reported moderate/severe psychological stress (PHQ-4) at both time points (28% and 29%, respectively). There was a small decrease (*P* = 0.001) in mean fatigue T-scores from 62.6 (SD 9.7) at S1 to 61.1 (SD 10.0) at S2, although these remain higher than the standardized US population score of 50 (SD 10).

**Table 3.** HRQL, mental wellbeing, stress and fatigue

	Survey 1 (Max *N* = 471)	Survey 2 (Max *N* = 302)	
In general, health now…	*n* (%)	*n* (%)	
Excellent	13 (3)	7 (2)	–
Very good	51 (11)	39 (13)	–
Good	137 (29)	95 (32)	–
Fair	177 (38)	113 (37)	–
Poor	93 (20)	48 (16)	–
Health-related quality-of-life	Mean (SD)	Mean (SD)	*P*-value (paired *t*-test)
SF-12 Physical Component Score (PCS)	38.0 (6.7)	38.8 (6.6)	*P* < 0.001
SF-12 Mental Component (MCS)	41.9 (5.2)	42.6 (5.0)	NS
EQ-5D-5L-VAS	61.5 (19.5)	63.8 (20.0)	*P* < 0.05
EQ-5D-5L utility index	0.66 (0.22)	0.68 (0.23)	NS
Warwick-Edinburgh Mental Wellbeing Scale (WEMWBS)	42.4 (10.2)	43.1 (10.5)	NS
Psychological stress (PHQ-4)	*n* (%)	*n* (%)	
Normal	190 (41)	118 (39)	–
Mild	146 (31)	98 (32)	–
Moderate	89 (19)	57 (19)	–
Severe	44 (9)	29 (10)	–
PHQ-anxiety	*n* (%)	*n* (%)	*P*-value (McNemar)
≤3	365 (78)	240 (79)	NS
>3	104 (22)	62 (21)	–
PHQ-depression			
≤3	376 (80)	247 (82)	NS
>3	93 (20)	55 (18)	–
			*P*-value (paired *t*-test)
Promis fatigue SF (*T*-score mean, SD)	62.6 (9.7)	61.1 (10.0)	*P* = 0.001

SF-12 Physical component score ranges from 0 to 100, with higher scores indicating better physical health functioning; EQ-5D-5L-VAS records self-rated health with minimum score of 0 (worst health) and maximum score of 100 (the best health you can imagine); EQ-5D health utility index scores were calculated with values are anchored at 1 (full health) and 0 (less than 0 is a state ‘worse than death’); Warwick-Edinburgh Mental Wellbeing Scale: minimum score of 14, maximum score of 70. The Scottish population mean score used for scale validation was 50.7; PHQ-4: Four Item Patient Health Questionnaire for Anxiety and Depression: minimum score of 0, maximum score of 12; scores are rated as normal (0–2), mild (3–5), moderate (6–8) and severe (9–12); PHQ-4 Anxiety component. Total score >3 suggests anxiety. PHQ-4 Depression component. Total score >3 suggests depression. Promis SF-V1-4A: Measure of Fatigue—A higher *T*-score represents more fatigue and mean standardized US population score is 50 (SD 10).

At S1, almost three-quarters (73%, *n* = 344) had contacted their GP practice; 38% (*n* = 158) contacted occupation health (OH), and 30% (*n* = 142) the NHS website, about their LC symptoms. Subsequent seeking of healthcare appears reduced at S2 (Table 2, available as Supplementary data at *Occupational Medicine* online). Thirty-four per cent (*n* = 158) had been to a hospital outpatient clinic and 47 (10%) had been admitted to hospital. At S2, 92 participants (31%) attended a new hospital clinic appointment with most of these ‘in-person’ (Table 2, available as Supplementary data at *Occupational Medicine* online). Overall, satisfaction with healthcare was low and 38% (*n* = 162) reported they were ‘dissatisfied’ with the healthcare received at S1 and 40% (*n* = 118) at S2 (Figure 1, available as Supplementary data at *Occupational Medicine* online).

During interviews, participant’s reported access to healthcare was limited. Early in the pandemic healthcare was perceived as closed for all but urgent care and participants were trying to self-manage their symptoms. By the second interview symptoms were not alleviating or getting worse, so all interviewees tried to access healthcare for diagnosis and treatment. Often there was no diagnosis or recognition of their LC or further follow-up by GP or another HCW. Others were referred to specialists but waiting times were long and often at the specialist appointment their referral symptoms were no longer their most pressing. For some individuals, access to specialist care was helpful in addressing specific symptoms but a holistic approach to their multiple and dynamic symptoms was preferred. The invisibility and the relapsing and remitting nature of a constellation of LC symptoms complicated diagnosis and support of LC. Participants commonly discussed feeling disbelieved, dismissed, forgotten, or abandoned by healthcare services, or there being a lack of meaningful help. A minority did have an understanding GP but there were limited treatment or referral options. Twenty-eight participants had a diagnosis of LC, though often brokered as possible, presumed or probable LC. Some participants had been given an alternative diagnosis (e.g. depression, anxiety or menopause). Many felt they had a better knowledge and understanding than their GP. Some engaged in persistently seeking healthcare and others felt options were limited, the single disease model of care did not fit their LC symptoms, and the NHS was overburdened. Table 4 presents supporting quotes.

Experiences with OH varied over time: poor at i1 (lengthy delays, poor knowledge and little help or support available); and mixed at i2 depending on health board (some with OH LC team). Current NHS OH policies (4-week phased return) were deemed not practical for dynamic and unpredictable conditions like LC.

Almost two-thirds had taken sick leave from work at both data collection points (S1: 65% (*n* = 306) and S2: 63% (*n* = 190)), and similar proportions (S1: 18% (*n* = 79) and S2: 17% (*n* = 48)) were unable to work due to LC symptoms. Concerns about their financial situation were similar at S1 and S2 (Figure 2, available as Supplementary data at *Occupational Medicine* online) with 49% worried about job security, 59% worried about their future financial situation and 49% needing to use savings to cover household bills.

Most participants were working (Interview 1 (i1): 37/50; Interview 2 (i2): 33/44; 9 off at both). By i2, seven participants felt they had nearly recovered but most were working with multiple and dynamic symptoms, with periods of improvement and exacerbation (often leading to sick leave) and others with debilitating symptoms. This affected their confidence in their abilities and professional identity. Those not working at both interviews reported the most severe symptoms with little improvement at i2 and all but one were doctors or nurses in high functioning roles. By i2, five had tried one or more attempts to return to work due to pressures (increased demand on NHS and colleagues, and feelings of guilt), which had exacerbated symptoms and necessitated further absence.

Over a third of our participants had changed their role or responsibilities at work; engaged in phased return; worked reduced hours and/or working from home, which enabled them to continue working when not experiencing an exacerbation. A third felt unsupported at work from HR, OH, line managers and/or colleagues. Support from colleagues and line managers was important for successful returns or remaining in work. Some had not disclosed their symptoms in i1 but by i2 had been selective in disclosure due to stigma with poor understanding of LC. They recognized their symptoms resulted in a reduced contribution at work and impacted upon their colleagues’ workload. All engaged in a range of self-management strategies to enable them to work, with work prioritized overall.

All those off work, or working in a reduced capacity, received a LC HCW payment, which ensured they received their pre-COVID salary but there was anxiety at i2 about how long this would continue and fear for their future. At i1, some had difficulty accessing this payment due to never having had a positive COVID test and/or LC diagnosis; however, by i2 salaried employees were in receipt where required. Independent contractors did not receive this payment and others had supplemented their pre-COVID salary with extra shifts, further impacting their anxiety and fear for the future and overall financial security.

## Discussion

All participants experienced various relapsing-remitting, changing and prolonged LC symptoms. The impact has been significant and devastating. Most HCWs in this mixed-methods study did not report significant improvements over time, with LC symptoms affecting their everyday life and ability to work. LC has been found to impact fatigue and HRQL more than some cancers [25]. Poor knowledge and support resulted in stigma, distress and despair. Participants feared for their future and financial security.

Sixty-four per cent of participants had a positive COVID test at first acute infection, compared with 59% in a UK LC lived experience study of 132 people [26] and 50% in a national population-based cohort study [27]. There was slight improvement in ability to undertake day-to-day activities, although a third continued to report these were ‘limited a lot’ at S2. Similar findings have been reported in a lived experience study where 43% were unable to return to usual activities [26], and 42% partially recovered in a national population-based cohort [27]. We found small increases/improvements in HRQL measures (Table 1) and statistically significant for physical health. However, mental wellbeing and overall HRQL were lower than published working-age populations and any changes/increases are lower than estimates representing minimally clinically important differences [28] indicating that our study population continued to experience poorer health overall [29].

Satisfaction with healthcare was low and participants engaged in self-management strategies to manage their symptoms, with work prioritized overall. They experienced stigma, distress, despair, grief for their former self and felt unsupported and abandoned by OH and the NHS at i1. By i2, as knowledge of LC grew, some participants described better understanding and support, illustrating length of follow-up for research is important [30]. Other research found 95% of survey respondents with LC experienced some form of stigma [31]. At i2, participants were anxious and feared for their future, prognosis, work and financial security. They also feared reinfection leading to relapses, vaccinations and unsuccessful returns to work. Fear around uncertainty was prevalent in other studies [32]. The harrowing stories of the devastating impact on people’s lives took an emotional toll on the researchers [33].

Returning to work in the NHS was a major challenge for many. This was often related to biopsychosocial problems, low pace of recovery and need for supportive strategies [34]. Concerns about ability to work and financial worries were common. A UK economic evaluation found LC significantly impacts productivity losses and provision of informal care, and a reduction in income due to LC symptoms [35]. Our study was conducted while NHS workers were still in receipt of the NHS special LC payment. The cessation of this payment will have a detrimental impact upon their financial stability [35].

Almost all who were off work at both interviews were doctors or nurses. High levels of fatigue and cognitive impairment were the most critical factors associated with reduced work capacity and influenced participants’ professional career in other studies [36]. Impact on leisure activities is also common [25]. Stigma and guilt associated with not working and the profound impact of LC on relationships, personal and professional relationships were also found by Callan *et al*. [37]. Furthermore, loss of identity has been a common finding in national and international literature [38].

Most of the participants had sought healthcare services for their LC symptoms, mainly from primary care and OH. Challenges in accessing and navigating healthcare are not unusual and in one report, only 3% accessed specialist services for LC [39]. Patients report feeling disbelieved and dissatisfied as they searched for physical mechanisms to explain their fluctuating symptoms [37]. In those with the most positive patient experiences, people felt listened to and that their often-bewildering experience was validated and affirmed with some describing ‘co-experting’ with healthcare professionals” [34]. Lack of access to these services may influence workability and hinder recovery.

We used mixed methods with longitudinal follow-up data collected after 6 months. As far as we are aware this is the first longitudinal mixed-methods study of LC and includes qualitative interviews (94 in total) with maximum variation sampling across professions [40]. This ensured a wide range of HCWs’ experiences were explored and provides unique and valuable insights of the impact of LC, including occupational and sociodemographic differences, symptoms and changes over time [10]. Participants were predominantly nurses, female and white; broadly reflecting sociodemographic characteristics of NHS workers in Scotland, and other reports of those with LC [10]. Survey respondents were recruited mainly through social media and there is no control group data: these limitations have been discussed previously [18].

Follow-up data collection was 6 months and there was uncertainty around the likely duration of LC symptoms at the time. Longer follow-up may have picked-up more substantial improvements [41]. Despite recruitment and sampling efforts, we had poor representation of those in ancillary posts and from BAME backgrounds. Other studies report 46% higher odds of LC in most deprived participants [42] and in those with migrant backgrounds [43]. Future research should seek to examine LC sufferers from these diverse backgrounds and with longer-term follow-up.

It is apparent that ongoing support and interventions will be needed for HCWs with LC to enable them to continue working in the NHS. These would aim to reduce sickness absence, improve return and retention in work and limit the impact on colleagues. Given the high prevalence of LC in the NHS and public sector, peer support could be advantageous [44]. Flexible and prolonged return-to-work policies and workplace adjustments which reflect the relapsing and remitting nature of LC could be beneficial but need rigorous evaluation. These would require support and adapting OH/HR policies in relation to LC and possibly other long-term conditions. Increased awareness of LC symptoms and experiences should be provided all NHS staff to improve knowledge, promote better understanding, improve care and reduce stigma.

Our study indicates that LC affects productivity, but it is important to note that most participants were stoically continuing to work in the NHS, which can be construed as presenteeism and presents a risk in safety critical roles. However, the impact of LC on colleagues’ workload is unknown. There is a need to protect the NHS workforce from COVID-19 infection and to understand the chronicity of LC with longer-term longitudinal studies. New treatments and clinical trials are needed, and in our experience, LC sufferers are highly motivated to be involved in research.

**Key learning points**

**What is already known about this subject:**

- Healthcare workers report a higher incidence of long COVID (LC) than other occupational groups. It is still unclear whether LC is a lifelong condition and if severity will persist.
- The NHS is experiencing a workforce crisis with a some of the shortfall predicted through sick leave, lower productivity or inactivity.

**What this study adds:**

- Multiple relapsing-remitting symptoms were common, and affected all aspects of daily activities, including ability to work.
- Any improvements in symptoms and overall health were small and they continued to report poorer health than in general working-age populations.
- Most participants were working in a reduced capacity and were fearful about their future ability to work, and financial security.

**What impact this may have on practice or policy:**

- Ongoing support and interventions will be needed for healthcare workers with LC to enable them to continue to work in the NHS; these would aim to reduce sickness absence, help them stay in work or return to work and limit the impact on colleagues.
- Flexible return-to-work policies and workplace adjustments which reflect the relapsing and remitting nature of LC could be beneficial but need rigorous evaluation; these would require support and adapting Occupational Health/Human Resources policies in relation to this and possibly other long-term conditions.

## Supplementary Material

kqae113_suppl_Supplementary_Table_S1-2_Figure_S1-2
