# Supplementary material for: Long COVID in healthcare workers: longitudinal mixed-methods study
Source: Occup Med (Lond). 2024 Nov 29;75(3-4):171–8. doi: 10.1093/occmed/kqae113 (PMC12257932; doi:10.1093/occmed/kqae113)
Supplement: kqae113_suppl_Supplementary_Table_S1-2_Figure_S1-2 [file kqae113_suppl_supplementary_table_s1-2_figure_s1-2.docx]

**Supplementary data**

**Table 1**. Symptoms of long COVID

|  | **Survey 1**  **(n=471)** | **Survey 2**  **(n=302)** |
| --- | --- | --- |
|  | **n (%)** | **n (%)** |
| Fatigue | 412 (87.5) | 221 (73.2) |
| ‘Brainfog’ | 374 (79.4) | 211 (69.9) |
| Breathlessness | 324 (68.8) | 163 (54.0) |
| Sleep disturbance | 254 (53.9) | 130 (43.0) |
| Heart palpitations | 227 (48.2) | 118 (39.1) |
| Headache | 220 (46.7) | 107 (35.4) |
| Joint pain | 216 (45.9) | 120 (39.7) |
| Muscle pain | 216 (45.9) | 107 (35.4) |
| Feelings of anxiety | 197 (41.8) | 103 (34.1) |
| Chest tightness | 195 (41.4) | 83 (27.5) |
| Post exertional malaise | 169 (35.9) | 98 (31.4) |
| Loss of taste/smell | 166 (35.2) | 69 (22.8) |
| Dizziness | 158 (33.5) | 70 (23.2) |
| Chest pain | 145 (30.8) | 68 (22.5) |
| Pins and needles; numbness in hands and feet | 139 (29.5) | 65 (21.5) |
| Cough | 128 (27.2) | 59 (19.5) |
| Symptoms of depression | 124 (26.3) | 65 (21.5) |
| Diarrhoea | 94 (20.0) | 33 (10.9) |
| Nausea | 92 (19.5) | 37 (12.3) |
| Tinnitus | 91 (19.3) | 45 (14.9) |
| Sore throat | 81 (17.2) | 50 (16.6) |
| Loss of appetite | 75 (15.9) | 24 (7.9) |
| Abdominal pain | 73 (15.5) | 39 (12.9) |
| Skin rashes | 64 (13.6) | 35 (11.6) |
| Fever | 63 (13.4) | 18 (6.0) |
| Earache | 54 (11.5) | 19 (6.3) |
| NO symptoms at S2 | n/a | 20 (6.6) |
|  | Mean (SD) [n] | Mean (SD) [n] |
| **Number of symptoms**  Mean, Median, or categorical | 9.47 (5.24) [468] | 7.14 (4.82) [302] |
|  |  |  |
| **Frequency of LC symptoms** | N (%) | N (%) |
| All of the time | 148 (31.6) | 67 (22.6) |
| Most of the time | 205 (43.7) | 106 (35.8) |
| Some of the time | 93 (19.8) | 90 (30.4) |
| Occasionally | 23 (4.9) | 26 (8.8) |
| None of the time | n/a | 7 (2.4) |

**Table 2.** Use of healthcare services, n (%)

|  | **Survey 1**  **Health services contacted about LC symptoms (n=471), n (%)** | **Survey 2**  **Since completed first survey, have you used/ contacted any of these services (n=302), n (%)** |
| --- | --- | --- |
| GP Practice | 344 (73.0) | 161 (53.3) |
| NHS 111 | 76 (16.1) | 7 (2.3) |
| Accident & Emergency | 43 (9.1) | 7 (2.3) |
| The NHS website | 142 (30.1) | 43 (24.2) |
| Occupational Health | 179 (38.0) | 90 (29.8) |
| None of the above | 92 (19.6) | 109 (36.1) |
| Other | 71 (15.1) | 38 (12.6) |

**Survey 1: Hospital appointments**

|  | **Attended in person** | **Telephone appointments** | **Video call appointments** |
| --- | --- | --- | --- |
| No. attending at least one appointment (%) | 154/471 (32.7) | 81 (17.2) | 33 (7.0) |
| No. of appts, median (IQR) [n] | 2 (1, 3) [143] | 2 (1, 3.5) [68] | 2 (1, 3) [22] |

**Survey 2. New Hospital outpatient clinic since previous survey**

|  | **Attended in person** | **Telephone appointments** | **Video call appointments** |
| --- | --- | --- | --- |
| No. attending at least one appointment (%) | 71/302 (23.5) | 48/302 (15.9) | 19/302 (6.3) |
| No. of appts, median (IQR) [n] | 2 (1, 2) [66] | 1 (1, 2) [43] | 1 (1, 2) [17] |

**Figure 1. Satisfaction with healthcare received for LONG Covid symptoms**

**Figure 2. Healthcare workers worries about their financial situation**
